# The Technique of Making a Porcelain Jacket Crown with Pressure*Read before the American Dental Society of Europe, April, 1912.

**Published:** 1912-10-15

**Authors:** J. O. Eppright

**Affiliations:** Cologne


### THE TECHNIQUE OF MAKING A PORCELAIN JACKET CROWN WITH PRESSURE.*

*Read before the American Dental Society of Europe, April, 1912.

BY DR. J. O. EPPRIGHT, COLOGNE.

Take a separating disc and reduce the proximal surfaces to the gum line to form a shoulder, and with small stones cut the labial and lingual surfaces as much as necessary in order to form a continuous shoulder at the gum margin; then take a sharp round excavating burr and cut the shoulder on the labial surface as far as possible under the gum, but care should be taken that the alveolar process be not interfered with. Then, with a diamond trephine and small carborundum points, finish the shaping of the tooth, so that its shape will be conical. Take an impression cup, which may be made of thin brass, on a Sharp's crown machine punch, the size of the tooth to be crowned, fill with soft impression compound and force it well under the gum and allow to thoroughly harden. With a sharp instrument cut the excess of compound away. Take a small impression cup made of ordinary tin plate and make an impression, including the teeth on each side of the tooth to be crowned. Care should be taken, in removing this impression, not to injure the impression of the tooth to be jacketed. Fill the latter with good hard cement, making the base of the model conical, and with a burr notch the cone; moisten with soapy water, mix more cement and fill the entire impression, making an exact model of the adjoining teeth. Then saw the impression nearly through, break it, and you have the model in three pieces. The model of the tooth is now free. Set the same in a swedger and swedge a platinum cap. I use the platinum foil which is specially prepared for inlay work. When you have the platinum cap swedged and trimmed, put your cement models together, take wax and make a model of the tooth to be crowned, and cast that either in tin or aluminum. Then fit it on the root of the tooth in the mouth and articulate. With tin foil enlarge the model about one-sixth of its own size. Make a mould of the tin model with cement, in a specially designed flask. Take this flask apart, trim the cement mould, and then, with a sharp burr, cut away the cement from the cutting edge of the tooth mould, leaving an opening through which the porcelain can be pressed home. The cement mould is now moistened sufficiently with soapy water, the flask put together with the tin model in position in the mould, and the model of the tooth is placed in the tin model which is in the mould. Fill the rest of the flask with the cement, which holds the model of the tooth in proper position in the flask. The flask is now taken apart. It has three parts, two containing the mould of the tooth and the third the model of the tooth. Place the platinum cap previously swedged in position on the tooth, line the cement mould with platinum foil such as is used for making the cap for the tooth. The mould for the porcelain is now complete. Mix the porcelain to the consistency of putty and then, in order to obtain your porper shades, take a shade ring, match the color of the tooth as near as possible and take a thin carborundum disc and cut the tooth on the shade ring, lengthwise; in half; thus observing the different colors of the porcelain on the shade tooth. Place the porcelain in the mould according to the colors you find in your shade tooth. Now put the flask together and place it in a swedger specially designed for this purpose and press it home by screwing. By introducing, then, soft, unvulcanized rubber you press the porcelain home in the mould with the screw press. Remove the flask and put it on a charcoal fire and biscuit the porcelain. After taking the flask asunder, take out the porcelain crown which has been biscuited and trim off all excess of porcelain and place in an electric oven and fuse to the desired gloss.—*Dental Record.*

